# Germline CNV Detection through Whole-Exome Sequencing (WES) Data Analysis Enhances Resolution of Rare Genetic Diseases

**DOI:** 10.3390/genes14071490

**Published:** 2023-07-21

**Authors:** Faidon-Nikolaos Tilemis, Nikolaos M. Marinakis, Danai Veltra, Maria Svingou, Kyriaki Kekou, Anastasios Mitrakos, Maria Tzetis, Konstantina Kosma, Periklis Makrythanasis, Joanne Traeger-Synodinos, Christalena Sofocleous

**Affiliations:** 1 Laboratory of Medical Genetics, St. Sophia’s Children’s Hospital, Medical School, National and Kapodistrian University of Athens, 11527 Athens, Greece; ftilemis@med.uoa.gr (F.-N.T.); dveltra@med.uoa.gr (D.V.); msvingou@med.uoa.gr (M.S.); kkekou@med.uoa.gr (K.K.); amitrakos@med.uoa.gr (A.M.); mtzetis@med.uoa.gr (M.T.); kkosma50@gmail.com (K.K.); pmakryth@med.uoa.gr (P.M.); jtraeger@med.uoa.gr (J.T.-S.); csofokl@med.uoa.gr (C.S.); 2 Research University Institute for the Study and Prevention of Genetic and Malignant Disease of Childhood, St. Sophia’s Children’s Hospital, National and Kapodistrian University of Athens, 11527 Athens, Greece; 3 Department of Genetic Medicine and Development, Medical School, University of Geneva, 1211 Geneva, Switzerland; 4 Biomedical Research Foundation of the Academy of Athens, 11527 Athens, Greece

**Keywords:** Whole-Exome Sequencing, Copy Number Variants, ExomeDepth, diagnostic yield, complex genotypes, rare diseases

## Abstract

Whole-Exome Sequencing (WES) has proven valuable in the characterization of underlying genetic defects in most rare diseases (RDs). Copy Number Variants (CNVs) were initially thought to escape detection. Recent technological advances enabled CNV calling from WES data with the use of accurate and highly sensitive bioinformatic tools. Amongst 920 patients referred for WES, 454 unresolved cases were further analysed using the ExomeDepth algorithm. CNVs were called, evaluated and categorized according to ACMG/ClinGen recommendations. Causative CNVs were identified in 40 patients, increasing the diagnostic yield of WES from 50.7% (466/920) to 55% (506/920). Twenty-two CNVs were available for validation and were all confirmed; of these, five were novel. Implementation of the ExomeDepth tool promoted effective identification of phenotype-relevant and/or novel CNVs. Among the advantages of calling CNVs from WES data, characterization of complex genotypes comprising both CNVs and SNVs minimizes cost and time to final diagnosis, while allowing differentiation between true or false homozygosity, as well as compound heterozygosity of variants in AR genes. The use of a specific algorithm for calling CNVs from WES data enables ancillary detection of different types of causative genetic variants, making WES a critical first-tier diagnostic test for patients with RDs.

## 1. Introduction

RDs have a global prevalence of 1 in 2000 people [1], and in approximately 80% an underlying genetic cause is recognized [2]. Although RDs are individually rare, 6000–7000 different RDs have been recognized to date (OMIM February 2023 updated) [3,4,5], and affect 400 million patients worldwide [6]. In general, RDs are heterogeneous conditions usually attributed to a broad range of pathogenic genetic variations, from the single-nucleotide level to chromosomal imbalances/rearrangements [7]. Among the categories of variants, CNVs are detected in ~10–20% of inherited human disorders, including Global Developmental Delay (GDD), Autism Spectrum Disorders (ASD), multiple congenital anomalies, immune deficiencies, several skin disorders, and other complex diseases [8,9,10].

Achieving molecular characterization of underlying mechanisms in RDs is often challenging, leading to daunting diagnostic odysseys [2,4,11]. Current procedures include detection of Single Nucleotide Variants (SNVs) by next generation sequencing methodologies, and of CNVs by array-comparative genomic hybridization (array-CGH), target-specific Multiplex Ligation-dependent Probe Amplification (MLPA), or Fluorescent in Situ Hybridization (FISH) [7,12,13]. WES has proven to be significantly valuable, reaching diagnostic yields between 30–50% higher than any other molecular genetic method so far [14]. Nonetheless, in about half of RD patients, a definitive molecular diagnosis may not be reached, for reasons that include limited experience with valid classification of SNVs and causative structural variants (SVs), so-far unknown underlying molecular-genetic mechanisms, and non-genetic aetiologies [15,16,17,18,19].

To further improve the diagnostic yield of WES, ancillary detection and analysis of CNVs from WES data has been established, based on a semi-quantitative analysis of sequencing depth coverage across the protein-coding regions analysed. One of the algorithms used is known as ExomeDepth, which calls CNVs by comparing the Depth of Coverage (DoC) between genomic regions of a test sample and a correlated set of reference samples [20]. This study reports further delineation of genetic variations with phenotypes, allowing characterization of diagnostic genotypes whilst concurrently minimizing the test cost to the patient/family and the time to a definitive diagnosis.

## 2. Materials and Methods

### 2.1. Patient Cohort

**Figure 1:**
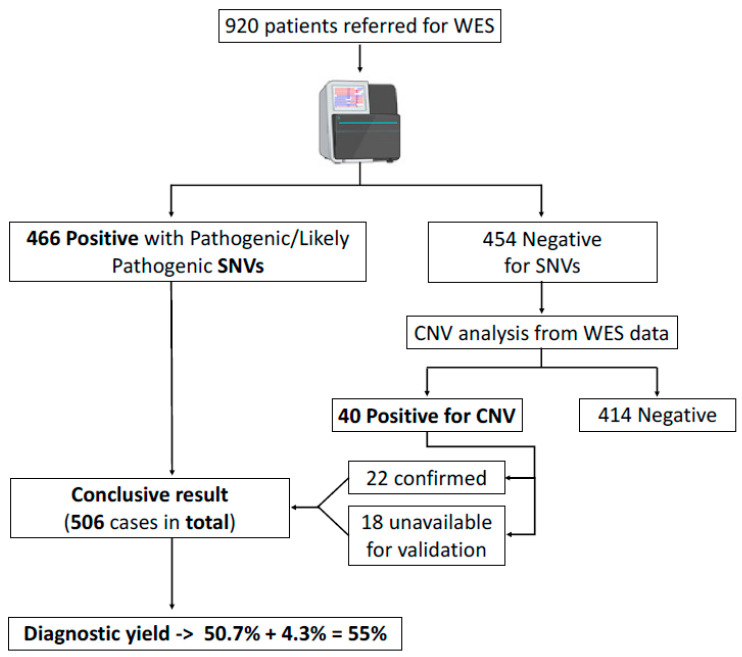
Workflow followed to result to a final diagnostic yield for WES.

**Figure 2:**
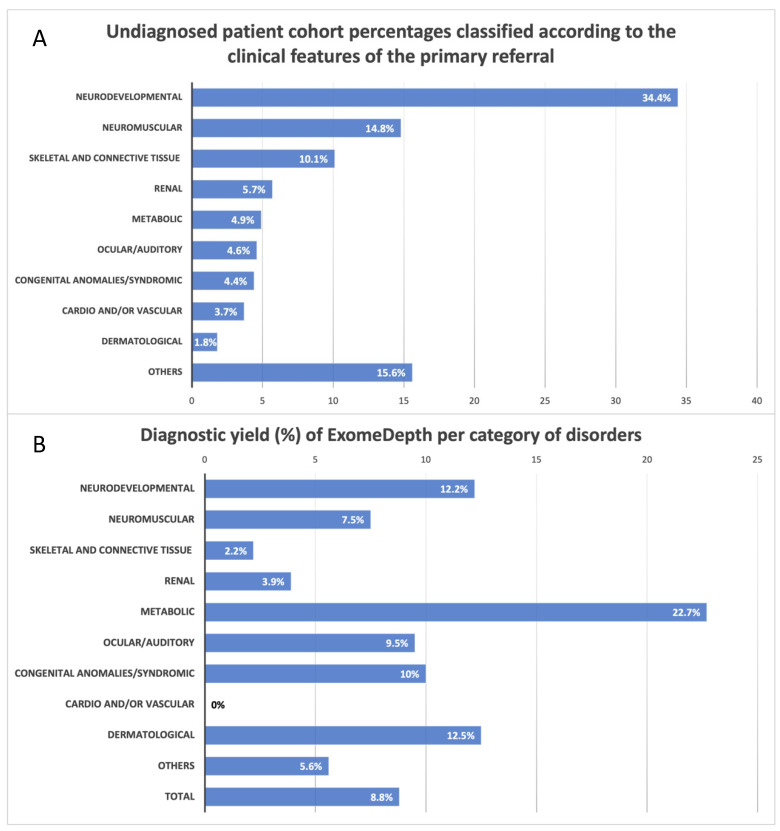
(**A**) Distribution of patients with respect to the clinical features of the primary referral. The cohort concerns patients remaining undiagnosed after WES analysis and the numbers are presented in percentages. (**Β**) Diagnostic yield (%) of the WES-based CNV algorithm (ExomeDepth) per category of disorders.

Four hundred and fifty-four (454), out of 920 cases referred to the Laboratory of Medical Genetics during the triennial 2020–2022, remained unresolved after WES analysis for SNVs, and were further assessed for CNVs using the ExomeDepth WES-based CNV-calling algorithm (Figure 1). The cohort was comprised of unrelated female (209) and male (245) patients, aged from 1 to 77 years old (83.5% were children and adolescents up to 18 years). The most common (156/454, 34.4%) reason for referral was neurodevelopmental abnormalities, followed by neuromuscular disorders (67/454, 14.8%) (Figure 2A, Table 1). For the remaining 231 (50.8%) cases, various clinical presentations were recorded and are categorized in Figure 2A and Table 1.

### 2.2. Whole-Exome Sequencing Data

Genomic DNA was extracted from peripheral blood lymphocytes according to standard methods. Singleton WES was performed in all cases using a Human Core Exome kit (Twist Bioscience, San Francisco, CA, USA) or IDT xGen Exome Research v2 kit (Integrated DNA Technologies, Coralville, IA, USA) and sequencing on an Illumina NextSeq 500 platform. Data were produced using BWA (hg19/GRCh37) and GATK algorithms [21,22] through an in-house bioinformatic pipeline or VarSome Clinical platform [23]. WES data quality acceptance metrics included a mean depth of coverage > 50×, with >97% regions at 20×. The SNV filtration process was based on a phenotype-driven strategy including: focus on a list of genes associated with the patient’s phenotype, using Human Phenotype Ontology (HPO) terms (https://hpo.jax.org/app/, accessed on 30 May 2023), Online Mendelian Inheritance in Man (OMIM; https://www.omim.org/, accessed on 30 May 2023), and in silico gene lists from the literature [18]. Subsequent variant classification followed the American College of Medical Genetics and Genomics (ACMG) guidelines [24].

### 2.3. CNV Analysis—ExomeDepth

Mining CNVs from WES data is enabled when applying variable algorithms, including ExomeDepth, cn.MOPS, and DeAnnCNV, to achieve the comparative analysis of distinct Depths of Coverage between different samples. To address possible pitfalls towards the detection of CNVs, all three algorithms were evaluated based on quantitative and qualitative parameters, such as the number and size of CNVs called among ten selected samples used as controls.

Overall CNV detection was based on the highly-rated R package ExomeDepth v1.1.15, with default settings [20], which uses ΒAΜ (Binary Alignment Map) files to call CNVs from WES pipelines. ExomeDepth is based on Depth of Coverage (DoC) comparisons between a test sample and a correlated reference set of samples (ideally 5–10). The test and the 10 reference samples (used in every run) come from the same batch, have the same gender, and do not include related individuals. In order to decrease a high false positive CNV calling rate and achieve more robust results, the required correlation was set above 0.97, and, to prioritize the most likely CNVs, two parameters were used: the Bayes Factor (BF = log10 of the likelihood ratio of data for the CNV call divided by the null-normal copy number) and the ratio of observed/expected number of reads. CNVs with BF < 10, and ratios of observed/expected number of reads > 0.8 for deletions and <1.1 for duplications, were filtered out. Further assessment used the ClassifyCNV v1.1.0 algorithm, which automatically implements the 2019 ACMG classification criteria [25]. CNV classification was evaluated according to the recommendations of the American College of Medical Genetics and Genomics (ACMG) and the Clinical Genome Resource (ClinGen) committees [26]. Finally, ancillary information was drawn from public databases and publicly available software, such as the ClinVar (https://www.ncbi.nlm.nih.gov/clinvar/, accessed on 30 May 2023), ClinGen (https://clinicalgenome.org/, accessed on 30 May 2023), DECIPHER (https://www.deciphergenomics.org/, accessed on 30 May 2023), and Franklin tools (https://franklin.genoox.com/clinical-db/home, accessed on 30 May 2023).

### 2.4. CNV Confirmation

Various procedures, including array-CGH, MLPA, gap-PCR, conventional karyotype, linkage, and segregation analysis of polymorphic short tandem repeat (STR) markers, and targeted RNA-sequencing, were performed to confirm copy-number change when possible (Table S1). The most appropriate CNV validation method was selected based on the size of the CNV, as well as the cost limitations and any time restrictions to achieve a diagnosis.

## 3. Results

### 3.1. CNV Detection and Characteristics

Table 2 presents detailed comparative analysis of data mined with ExomeDepth, cn.MOPS, and DeAnnCNV when employed for ten cases of reference. All findings were assessed with additional orthogonal methods for confirmation and validation. As shown, ExomeDepth was proven to have the highest performance, efficacy, and accuracy, and was selectively applied for the purposes of the cohort and the analysis of 454 cases.

For each case, some 200–250 CNVs, concerning all chromosomes, were called before being subjected to filtration. In terms of CNV characteristics, deletions exceeded duplications (Table S2), since, by default, read count methods are more accurate towards the detection of deletions [27,28], and sizes ranged from single exons to several Mbs.

A total of 40 highly scored known or novel CNVs in phenotype-relevant genes and/or chromosomal regions were detected, comprising 33 (82.5%) deletions and 7 (17.5%) duplications (Table S3 and Table 3 and Figure 1). Following ACMG and ClinGen recommendations, 24 were classified as pathogenic, 15 as likely pathogenic, and 1 as a Variant of Uncertain Significance (VUS). In respect to the mode of inheritance, 20 CNVs were characterized as autosomal dominant (AD) and 13 as autosomal recessive (AR), whereby 2 were homozygous for a deletion and 11 compound heterozygotes of the CNV and a pathogenic/likely pathogenic SNV (Table 3 and Table S3). Five CNVs concerned the X chromosome (X-linked, XL), while two aneuploidies were also recorded. With respect to size, the CNVs detected included alterations restricted to only 1 or up to multiple exons of the same gene, an entire gene, multiple contiguous genes, and even entire chromosomes, including well characterized trisomies such as Klinefelter syndrome (47, XXY) and Down syndrome (Trisomy 21–47, XX, +21) (cases 35 and 279 respectively). Of note, patient 279 was already diagnosed with Down syndrome by conventional karyotype, but was referred for WES due to additional unrelated findings such as anaemia, airway obstruction, and bronchiolitis obliterans (Table S4).

Five CNVs were considered as novel, with no previous records in the literature and the databases (ClinVar, ClinGen). Four of these were associated with neurodevelopmental disorders, and one with skeletal/connective tissue abnormalities (patients 9, 10, 11, 14, and 25). Additional information about these novel CNVs and corresponding patients is summarized in Table 3.

The accuracy and reliability of ExomeDepth was evaluated for 22/40 CNVs (cases) using an alternative method, such as array-CGH or MLPA. The presence of CNVs was confirmed and further processed to demonstrate a high concordance (>90% overlap) of sizes and breakpoints estimated by ExomeDepth and array-CGH or/and MLPA (Supplementary Materials, Table S1).

### 3.2. Diagnostic Yield

From amongst 920 patients referred for WES, 466 (50.7%) received a diagnosis after the detection of a pathogenic/likely pathogenic SNV, and 40 following further assessment and exclusive detection of CNVs by ExomeDepth [18,19]. Enriched by 4.3%, a total of 506 cases were ultimately resolved, supporting an accumulated diagnostic yield for WES of 55%. The isolated diagnostic yield of WES-based CNV analysis was 8.8% (40/454). With respect to separate disease categories, CNVs were abundant in metabolic disorders (22.7%, 5/22), followed by skin abnormalities (12.5%, 1/8) and neurodevelopmental disorders (12%, 19/156) (Table 1, Figure 2B). Of note, almost half of the CNVs detected (47.5%, 19/40) concerned disorders of neurodevelopment (Table 1).

On the other hand, 414/920 (45%) patients still remain unresolved, as no causative CNVs or SNVs were identified. More specifically, and with respect to separate disease categories, CNVs were scarce in cases with renal abnormalities (3.9%, 1/25), skeletal and connective tissue disorders (2.2%, 1/46), and cardio and/or vascular abnormalities (0%, 0/17) (Table 1, Figure 2B).

## 4. Discussion

CNVs represent genomic regions with variable copy numbers throughout the genomes of different individuals, including both amplifications and deletions of DNA sequences. CNVs can result in no or minimal phenotypes, or substantial effects on health [29]. The gold standard for routine CNV detection in genetic diagnosis settings includes a genome wide array-CGH or SNP microarray [7,30,31], addressing large CNVs (from 50 Kbs to several Mbs), or, when targeting specific regions of interest, MLPA [7]. In the context of time and cost-savings, NGS-based CNV detection approaches are developed to bridge the gap and allow the discovery of variable CNVs ranging in size from 200 bases to several Mbs. The base-by-base view of genomic regions provided by NGS may facilitate the recognition of small and/or novel CNVs, which may escape a-CGH and MLPA detection, as well as mapping their exact location, albeit with ambivalent sensitivity, specificity, and false discovery rates.

Widely available CNV callers, including the highly-cited ExomeDepth, cn.MOPS, and DeAnnCNV, are generally characterized as user-friendly, fairly accurate, and less prone to false identifications, and can detect a wide spectrum of CNV sizes [32,33]. Following a rough assessment of ten cases of reference with all three algorithms, ExomeDepth was proven to have the highest performance, efficacy, and accuracy. More specifically, ExomeDepth detected both small (<1 Kb, even at the level of an exon) and large variations (at the chromosome level, e.g., Trisomy 21 or Klinefelter syndrome), including all CNVs in the ten cases, and with a high concordance (>90%) to the estimated breakpoints as compared to those given by orthogonal methods. Furthermore, ExomeDepth and cn.MOPS, although first published nearly a decade ago, are automatically updated almost every year (last updates on 2 November 2022 and 10 July 2023, respectively). Regarding cn.MOPS, advantageous “user-friendliness” allowed the simultaneous analysis of multiple BAM files using a single command; however, with a lower accuracy in respect to both the failure to detect one (1/10) variation and the ability to precisely define breakpoints and sizes of the nine CNVs identified. As for DeAnnCNV, it is an easy-to-use online tool, which, although easily implemented with no commands, fails to process BAM files, thereby requiring additional tools (e.g., PrepocessFiles) and further commands to convert files to the desired format (.tar.gz). Finally, DeAnnCNV achieved diagnosis in only 50% of the control samples, probably due to the very small number of CNVs (2–3) called in total for each case. Findings from the comparative analysis ruled in favour of ExomeDepth as the most appropriate tool for the detection and characterization of CNVs from WES data.

Implementation of the ExomeDepth bioinformatic tool promoted effective identification of phenotype-relevant CNVs, thus increasing the diagnostic yield of WES. Forty clinically relevant CNVs, ranging in size from one exon up to an entire chromosome (Table 3; Supplementary Materials, Tables S3 and S4) allowed the elucidation of unresolved cases, whereby expanding the final diagnoses of WES by 4.3%. The overall diagnostic yield in this cohort of patients reached 55%, considerably higher of that achieved with the exclusive detection of SNVs [15,16,17,18]. CNVs in strongly phenotype-relevant genes/chromosomal regions and with good quality metrics (BF, reads ratio) were primarily examined and further evaluated, minimizing false positive results. In terms of diagnoses restricted to ExomeDepth findings, this reached 8.8% (40/454 of patients), with CNVs being most commonly detected in neurodevelopmental disorders, metabolic abnormalities, and neuromuscular disorders (Table 1). Around 10–20% of the intellectual development disorders are attributed to CNVs, so far traditionally detected by array-CGH [34,35,36,37]. However, recognition of small (<30 Kb) CNVs depends on the resolution of each platform and may be missed, indicating that ancillary analysis of WES data for the detection of both known and novel SNVs and CNVs may be appropriate when investigating patients with neurodevelopmental disorders.

Accurate confirmation of 22/40 CNVs (Supplementary Materials, Table S1) with array-CGH and MLPA indicated a high concordance (overlap > 90%) of estimated breakpoints, with the exception of a sole CNV (patient 15) where discordance may be attributed to limited WES coverage in non-overlapping regions. CNVs not eligible for validation include those that escape other diagnostic procedures, such as array-CGH and MLPA, due to small sizes, or a lack of either array or MLPA targets.

Among the advantages of calling CNVs from WES data, characterization of complex genotypes comprising both CNVs and SNVs or small insertions/deletions minimizes costs and time to final diagnosis [38,39,40], while also allowing differentiation between true or false homozygosity, as well as compound heterozygosity of variants in AR genes. In point of fact, 11 patients with pathogenic SNVs in AR genes were falsely characterized as either homozygous for an SNV (patients 14, 25, 30, and 40), when they actually carried a large deletion *in-trans* configuration with the SNV (Table 3; Supplementary Materials, Table S3), or as carriers (patients 8, 10, 20, 21, 23, 29, and 32), while they really were compound heterozygotes for the SNV and a CNV in the same AR gene (Table 3; Supplementary Materials, Table S3).

Previously undescribed CNVs called from WES data require further investigation towards final classification. Five novel CNVs were detected in patients 9, 10, 11, 14, and 25 (Table 3). Patient 9, a 5-year-old male, presented with delayed and impaired speech and apraxia; a novel duplication was detected, including the *SH3KBP1* gene (also called *CIN85*), which encodes an 85-kDa CBL-interacting protein (CIN85) that facilitates protein-protein interactions [41]. Previous findings of *SH3KBP1* pathogenic lesions were reported in patients with the X-linked recessive primary Immunodeficiency-61 (IMD61) [42], characterized by recurrent infections in early childhood and accompanied by neurodevelopmental deficits, including attention deficit hyperactivity disorder (ADHD) and impaired adaptivity. Patient 9 presented with no immunodeficiency or any other immune system abnormality, but these may manifest later in life. Functional studies on the role of CIN85 indicated that this regulator of endocytosis in neurons, which presents with functional similarities to immunological synapses in T cells, may well be involved in behaviour abnormalities [43]. In line with other well-established findings—including that of chromosomal region 22q11.2, which is well acknowledged to result in DiGeorge heart failure syndrome when deleted, but results in a variable neurodevelopmental disorder with no cardiological problems when duplicated—a similar hypothesis may be instigated. Loss of function variants in *SH3KBP1* are most commonly detected in patients with IMD61, but findings on two families with intellectual disability and large duplications encompassing *SH3KBP1*, *EIF1AX,* and *RPS6KA3* genes indicate a possible role for *SH3KBP1* in addition to that of *RPS6KA3* [44,45]. Limited data hinder further explanation on how phenotypic findings may be attributed to duplications which call for additional assessments and studies [42].

Patient 10, referred due to seizures (since birth), GDD, and skeletal abnormalities, is a compound heterozygote of *PNPO* variants, comprising a pathogenic SNV and a large novel duplication encompassing the *PNPO* gene (Table 3). The detected PNPO p.(Arg225His) variant is a known recurrent pathogenic variant that disrupts protein function; duplications of *PNPO*, although recorded in decipher (Decipher database, #293487), are still under investigation to allow resolution of underlying mechanisms (gene dosage imbalance, possible excess protein aggregation, disruptions of promoters, or other important for transcription binding sites, etc.). Biallelic *PNPO* pathogenic variants are related to 5′-phosphate oxidase (PNPO) deficiency, and diagnosis may be supported by the measurement of PNPO enzyme activity, which, although recommended, was finally refuted by the family of patient 10. PNPO deficiency is characterized by variable types of seizures, typically resistant to most antiepileptics; however, irrelevant of the type and onset of seizures, life-long treatment with B_6_ vitamer pyridoxal 5′-phosphate (PLP), or pyridoxine (PN), may be relieving and protective of seizure complications showcasing the importance of early diagnosis. Notably, skeletal anomalies such as polydactyly, which are not directly related to PNPO deficiency, may be attributed to one of the other genes within the duplication.

Patient 11, a 7-year-old female with GDD, inferred mobility, and dysmorphic features, carries a novel deletion on chromosome 4, encompassing 11 genes (Table 3). Although deletions of 4p14-p13 region are not as-yet associated with specific clinical entities, duplications leading to complete or partial trisomy 4p present with characteristic craniofacial malformations, growth retardation, and impaired neurodevelopment. Further in the same context, the recent publication of a larger (2.1 Mb) de novo deletion (DEL:chr4:39909986_42050575) in a patient with a specific learning disability and facial deformities (Decipher database, #345528) is supportive of a specific role for this region in neurodevelopment.

WES analysis allowed characterization of compound heterozygosity for *WDR73* variants comprising a small likely pathogenic duplication and a large novel deletion, encompassing the entire *WDR73* gene (Table 3) in patient 14. Pathogenic or likely-pathogenic variants of *WDR73* are linked to infantile-onset cerebellar atrophy (CA), and the rare autosomal recessive Galloway–Mowat syndrome (GMS), characterized by microcephaly and brain anomalies, such as CA, intellectual disability, and highly heterogeneous, in respect of both severity and age of onset, renal manifestations [46]. Patient 14 is a 15-year-old female referred for seizures, GDD, microcephaly, and corpus callosum and cerebellar atrophy, all compatible with *WDR73* deficiency and CA or GMS, even in the absence of any renal issues.

In patient 25, a 3-year-old girl presenting with polydactyly, brachydactyly, and hypoplastic teeth, WES revealed compound heterozygosity of *IQCE* variants; a small deletion and a novel large deletion, encompassing exons 2–18 [39].

Finally, patient 17, a 33-year-old male referred for GDD accompanied by muscle dysfunction and additional variable symptoms, carries a large heterozygous deletion on chromosome 8, involving 60 genes (Table 3). Microdeletions of a specific 8q21.3–q22.1 region (~3 Mb) have been linked with Nablus mask-like facial syndrome (NMLFS, MIM#608156). NMLFS is characterized by distinctive facial features, including blepharophimosis, tight-appearing, glistening facial skin, distinctive ears, and a happy demeanour [47]. However, patients such as patient 17, in whom larger deletions encompass the NMLFS critical region, do not exhibit the cardinal facies of NMLFS [48,49]. Of note, the myopathy and muscle weakness recorded in patient 17 have never been described before in similar cases, and may not be attributed to the deletion, indicating that some other genetic factors escaping the WES diagnosis may be involved.

ExomeDepth has proved one of the most sensitive, widely applied algorithms used to call CNVs from WES data [12,13,28,50,51]. As a robust and user-friendly application (only moderate R programming skills are required), ExomeDepth is appropriate for almost any laboratory handling WES data. However, despite well acknowledged advantages of WES-based CNV analysis, considerable limitations, including a high false positive rate, the need for batched and gender-matched analysis of samples, and additional homogeneous coverage of sequencing reads, restrict its inclusion as a gold-standard method for CNV detection [27]. In addition, complete characterization of CNVs to include breakpoints, especially if in non-coding regions, is limited, and intergenic and/or intronic CNVs escape detection [7,27,52]. Nevertheless, as sequencing libraries, capture kits, and bioinformatic pipelines are continuously upgraded, such restrictions are expected to diminish [53]. Future routine applications of currently-costly third-generation sequencing (TGS) methods are also anticipated to address many limitations and provide further possibilities in SV detection (mainly due to long-read sequencing) [54,55].

## Figures and Tables

**Table 1:** Number of resolved and unresolved patients after analysis of WES data for CNVs.

Category of Disorder	Cases	Considered Resolved after CNV Analysis	Remained Unresolved after CNV Analysis	CNV Positive Rates (Enriched Diagnostic Yield *)
Neurodevelopmental	156	19	137	12.2% (4.2%)
Neuromuscular	67	5	62	7.5% (1.1%)
Skeletal and connective tissue	46	1	45	2.2% (0.2%)
Renal	26	1	25	3.9% (0.2%)
Metabolic	22	5	17	22.7% (1.1%)
Ocular/Auditory	21	2	19	9.5% (0.4%)
Congenital anomalies/Syndromic	20	2	18	10% (0.4%)
Cardio and/or vascular	17	0	17	0% (0%)
Dermatological	8	1	7	12.5% (0.2%)
Others	71	4	67	5.6% (0.9%)
Total	454	40	414	8.8% (4.3%)

* Enriched diagnostic yield represents the increase in diagnosis among the total cohort (920 patients).

**Table 2:** Comparative analysis of algorithms applied to call CNVs from WES data of 10 selected cases of reference.

Algorithm/ Tool	Total Number of CNVs Detected in Each Sample	Number of Causative CNVs Identified (10 Positive Controls)	Concordance between Algorithms and Confirmation Methods	Range of CNVs Sizes	Need for Additional Tools?
ExomeDepth	140	10 (of 10)	96%	1 exon—Entire chromosome	No
cn.MOPS	35	9 (of 10)	85%	3 exons—Entire chromosome	Yes—for annotation
DeAnnCNV	4	5 (of 10)	91%(in the 5 detected)	3 exons—Some Kbs	Yes—to convert BAM files to .tar.gz format

**Table 3:** List of patients with novel CNVs detected by ExomeDepth from WES data.

Patient	Clinical Features	Age/Gender	Type	CNV Coordinates in Grch37/hg19 (CNV Size)	BF	Reads Ratio	Genes	No of Coding Genes/Exons (Exon No)	CNV Classification (ACMG/Clingen Score)	CNV Confirmation Method	Inheritance	SNV Combined with the CNV	Disease (MIM Number)
Neurodevelopmental Disorders													
9	Delayed speech and language development	5-years-old/M	DUP	chrX:19564040_19954016 (390 Kb)	124	1.64	*SH3KBP1* (NM_031892)	15 Exons (1–15)	VUS: 0 (1A, 3A, 4N, 4O)	aCGH: arr[GRCh37] Χp22.12(19,591,222_19,935,900)x2	XL	n/a	Immunodeficiency 61 (300310)
10	Seizures, GDD, preaxial hand polydactyly, knee dislocation, scoliosis and hypertelorism	11-years-old/F	DUP	chr17:44949883_46507482 (1.6 Mb)	1740	1.4	*PNPO* (Whole gene) + 34 genes	35 genes	LIKELY PATHOGENIC: 0.9 (1A, 2H, 2K, 2L, 3A, 4L, 4O)	STRs (Duplication Paternal) + Sanger (SNV Maternal)	AR, Compound HTZ with Pathogenic SNV	PNPO: c.674G>A, p.(Arg225His)	Pyridoxamine 5′-phosphate oxidase deficiency (610090)
11	GDD, dysplastic corpus callosum, inability to walk, almond-shaped palpebral fissure	7-years-old/F	DEL	chr4:40337485_41941400 (1.6 Mb)	342	0.7 (HTZ)	*NSUN7*, *UCHL1*, *CHRNA9*, *MIR4802*, *APBB2*, *TMEM33*, *PHOX2B* + 4 genes	11 Genes	PATHOGENIC: 1 (1A, 2A, 2H, 3A, 4L)	n/a	AD (Haploinsufficiency)	n/a	n/a
14	Seizures, tetraplegia, GDD, microcephaly, corpus callosum and cerebellar atrophy, reduced cerebral white matter volume, cataract and hip dislocation	15-years-old/F	DEL	chr15:84908070_85681134 (773 Kb)	690	0.55 (HTZ)	*WDR73* + 12 genes	13 Genes	PATHOGENIC: 1 (1A, 2A, 2H, 3A, 4L, 4N)	n/a	AR, Compound HTZ with Pathogenic SNV (seemed HOM)	*WDR73*: c.525_565dup, p.(Asp189Valfs*6)	Galloway-Mowat syndrome 1 (251300)
Skeletal/Connective tissue Disorders													
25	Polydactyly, brachydactyly and hypoplastic teeth	3-years-old/F	DEL	chr7:2606751_2641098 (34.3 Kb)	29	0.7 (HTZ)	*IQCE* (NM_152558)	17 Exons (2–18)	LIKELY PATHOGENIC: 0.9 (1A, 2B, 2E, 3A)	STRs (Deletion Maternal) + Sanger seq for SNV (SNV Paternal)	AR, Compound HTZ with Pathogenic SNV (seemed HOM)	*IQCE*: c.895_904del, p. (Val301Serfs*8)	Polydactyly, postaxial, type A7 (617642)

F, female; M, male; BF, Bayes factor; GDD, Global Developmental Delay; DEL, deletion; DUP, duplication; Kb, kilobase pair; Mb, megabase pair; HOM, homozygous; HTZ, heterozygous; VUS, Variant of Uncertain Significance; aCGH, array-comparative genomic hybridization; STR, Short Tandem Repeats; SNV, Single Nucleotide Variant; AD, autosomal dominant; AR, autosomal recessive; XL, X-linked; n/a, not applicable.

## Data Availability

Findings of this study are submitted in the ClinVar database (Accession number: SUB13480173). Further supportive data are available upon reasonable request from the corresponding author and may not become publicly available due to privacy or ethical restrictions.

## Supplementary Materials

The following supporting information can be downloaded at: https://www.mdpi.com/article/10.3390/genes14071490/s1, Table S1: Concordance of CNVs detected between ExomeDepth and confirmation methods; Table S2: Characteristics of all the CNVs identified by ExomeDepth in 454 samples; Table S3: List of patients with CNVs detected by ExomeDepth from WES data; Table S4: Whole chromosomal duplications detected by ExomeDepth in two patients referred for WES.
