# Supplementary material for: Germline CNV Detection through Whole-Exome Sequencing (WES) Data Analysis Enhances Resolution of Rare Genetic Diseases
Source: Genes (Basel). 2023 Jul 21;14(7):1490. doi: 10.3390/genes14071490 (PMC10379589; doi:10.3390/genes14071490)
Supplement: Supplementary file 1 [file genes-14-01490-s001.zip › Table S1.pdf]

Table S1. Concordance of CNVs detected between ExomeDepth and confirmation methods.

Patient	Type	CNV predicted coordinates (Grch37/hg19)	CNV size	Zygosity	GENES	CNV Confirmation Method	Additional Information	Similarity to confirmation methods
3	DEL	chr12:11463270_14019142	2.6 Mb	0.67 (HTZ)	*GRIN2B* + 19 genes	MLPA: MRC P410-A2 - *GRIN2B* Deletion	MRC P410-A2 MLPA kit	100%*
6	DUP	chr15:23684691_28566579	4.9 Mb	1.21	*GOLGA6L2, MKRN3, MAGEL2 + 11 genes*	MS-MLPA: MRC ME028-D1 + STRs	MRC ME028-D1 MS-MLPA kit	n/a
							STR - *GABRB3* Primers: 5' FAM-CTCTTGTTCCTGTTGCTTTCAATACAC-3' (forward) and 5'-CACTGTGCTAGTAGATTCAGCTC-3' (reverse)	
8	DEL	chr16:8841965_8851663	9.7 Kb	0.51 (HTZ)	*ABAT*	Targeted RNA Sequencing: deletion exons 4-6 and Sanger seq for SNV. In trans: Deletion Maternal and SNV Paternal	Targeted RNA Sequencing - ABAT exons 2-8 Primers: 5'-ATGGCCTCCATGTTGCT-3' (forward) and 5'-CCGGTACCACATGAAGAT-3' (reverse)	n/a
							Sanger seq for SNV - *ABAT* exon 14 Primers: 5'-GCCCACTAGATTAGTTTCTCTCC-3' (forward) and 5'-CCGTTACGAACTGCTGGAA-3' (reverse)	
9	DUP	chrX:19564040_19954016	390 Kb	1.64	*SH3KBP1*	aCGH: arr[GRCh37] Xp22.12(19591222_19935900)x2	4x180K G3 CGH+SNP microarray platform (G4890A, Agilent Technologies)	88.5%

10	DUP	chr17:44949883_46507482	1.6 Mb	1.4	*PNPO* (Whole gene) + 34 genes	STRs (Duplication Paternal) + Sanger (SNV Maternal)	STRs - In duplicated region: a) 16xCA Primers: 5' FAM-CACTCAGCCTATACATGCTTCT-3' (forward) and 5'-TCTTAAGTTTAGCAACTCCCTTCA-3' (reverse), b) 19xAC 5' FAM-ATCGCATGACTGCACTTC-3' (forward) and 5'-GACCTTGAGGATTGGCTTT-3' (reverse), c) 21xAC 5' FAM-GTCCTGTCAACGTGATAGATAAA-3' and 5'-CTAAGGAGCTTGGACTTCATT-3' (reverse)<br>Sanger seq for SNV - *PNPO* exon 7 Primers: 5'-GCAACTTCCTTCAAGAGG-3' (forward) and 5'-AGGGAAATGGGTTGAGTTTAG-3' (reverse)	n/a
12	DEL	chr7:72717395_74173168	1.5 Mb	0.53 (HTZ)	*ELN, LIMK1 + 23 genes*	MLPA: MRC P245-A2 MLPA Analysis. Deletions on *ELN* and *LIMK1* index genes confirmed the presence of the large deletion on chromosome 7q11.23	MRC P245-A2 MLPA kit	n/a
13	DEL	chrX:153295818_153298008	2.2 Kb	0.69 (HTZ)	*MECP2*	MLPA: MRC P015-F2 - *MECP2* Deletion, Exons 2-3	MRC P015-F2 MLPA kit	100%*
15	DUP	chr1:146465878_147416212	950.3 Kb	1.28	*NBPF14, PRKAB2, FMO5, CHD1L, BCL9, ACP6, GJA5, GJA8, GPR89B*	aCGH: arr[GRCh37] 1q21.1q21.2(146507518_147824207)x3	4x180K G3 CGH+SNP microarray platform (G4890A, Agilent Technologies)	73%
16	DEL	chr2:166872248_167334216	462 Kb	0.48 (HTZ)	*SCN1A, SCN9A, SCN7A*	MLPA: MRC P137-C1 - *SCN1A* Deletion, Exons 1-16	MRC P137-C1 MLPA kit	100%*

17	DEL	chr8:94713453_97978274	8.8 Mb	0.69 (HTZ)	*MMP16, NBN, DECR1* + 35 genes	aCGH: arr[GRCh37] 8q21.3q22.1(89148404_98017865)x1	4x180K G3 CGH+SNP microarray platform (G4890A, Agilent Technologies)	98.8%
22	DEL	chrX:31950355_32305635	355.3 Kb	0 (HEM)	*DMD*	MLPA: MRC P034-B2 and P035-B1 - *DMD* Deletion, Exons 44-45	MRC P034-B2 + P035-B1 MLPA kit	100%*
24	DEL	chrX:149761077_149818374	57.3Kb	0 (HEM)	*MTM1*	MLPA: MRC P309 - MTM1 Deletion, Exons 2-10	MRC P309 MLPA kit	100%*
25	DEL	chr7:2606751_2641098	34.3 Kb	0.7 (HTZ)	*IQCE*	STRs (Deletion Maternal) + Sanger seq for SNV (SNV Paternal)	STR - *IQCE* Intron 8 19xGT Primers: 5' FAM - AAGTAGATGGTGAAAGCCTAGC-3' (forward) and 5'-TGAGCTGAATGCTGCAGTG-3' (reverse)<br>Sanger seq for SNV - *IQCE* exon 12 Primers: 5’-ATTCACCTGTCCCTCGTTTC-3’ (forward) and 5’-TTGCCCAAGGGTTCACATC-3’ (reverse)	n/a
26	DEL	chr6:491126_1624775	1,1 Mb	0.62 (HTZ)	*FOXC1, FOXF2, FOXQ1, GMDS, EXOC2, HUS1B*	aCGH: arr[GRCh37] 6p25.3(510688_1671743)x1	4x180K G3 CGH+SNP microarray platform (G4890A, Agilent Technologies)	91.6%
27	DEL	chr17:34842545_36293050	1,5 Mb	0.65 (HTZ)	*HNF1B* (Whole gene) + 14 genes	MLPA: MRC P463-A2 - *HNF1B* Deletion	MRC P463-A2 MLPA kit	100%*

29	DEL	chr5:133956623_133959709	3.1 Kb	0.32 (HTZ)	*SAR1B*	STRs (Deletion Maternal) + Sanger seq for SNV (SNV Paternal)	STR - SAR1B Intron 2 10xATTT Primers: 5' HEX-GGCATTGTAGATATGTTAAGGCAAA-3' (forward) and 5'-GAGGTAAGGAGTTTGACACCAG-3' (reverse)<br>Sanger seq for SNV - *SAR1B* exon 7 Primers: 5'-AGGAGGCTTCTTGTGTGTTC-3' (forward) and 5'-GCATGTTGAGCAATCAAATCTCT-3' (reverse)	n/a
30	DEL	chr9:97401423_97401592	170 b	0.52 (HTZ)	*FBP1*	GAP-PCR: *FBP1* Deletion:chr9:97396231_97401641 (5410bp), identical with PMID: 27101822	GAP-PCR *FBP1* Primers (deletion 5410bp, including exon 1 and part of intron 1): 5'-TAAAGGTTTCCGCGATTCAC-3' (forward) and 5'-GCTAGGACCAGCGATTTCAG-3' (reverse)	n/a
31	DELE	chr18:55020078_56892966	1.9 Mb	0.52 (HTZ)	*ATP8B1, ST8SIA3, ONECUT2, FECH, NARS1, NEDD4L, ALPK2, MALT1, ZNF532, SEC11C, GRP*	aCGH: arr[GRCh37] 18q21.31q21.32(54919098_56963819)x1	4x180K G3 CGH+SNP microarray platform (G4890A, Agilent Technologies)	93.1%
32	DEL	chr1:216138660_216270555	131,9 Kb	0.66 (HTZ)	*USH2A*	MLPA: MRC P362-A3 MLPA Analysis - *USH2A* Deletion, Exons 22, 24, 26, 28, 30, 32, 34	MRC P362-A3 MLPA kit	100%*
35	DUP	chrX:200855_155240074	155 Mb	1.8	Additional X chromosome	Conventional Karyotype	n/a	100%

37	DEL	chrX:105066840_106486528	1.4 Mb	0.00873 (HEM)	*PIH1D3, TBC1D8B, SERPINA7* + 9 genes	aCGH: arr[GRCh37] Xq22.3(105088238_ 106606637)x0	4x180K G3 CGH+SNP microarray platform (G4890A, Agilent Technologies)	93.3%
38	DEL	chr22:22893189_24177119	1.3 Mb	0.67 (HTZ)	*SMARCB1* (Whole gene) + 16 genes	MLPA: MRC P258-C2 MLPA Analysis - *SMARCB1* Deletion	MRC P258-C2 MLPA kit	100%*

* These percentages (%) are referred only to the exons of the phenotype-relevant gene analyzed by MLPA, NOT to the entire CNV coordinates detected by ExomeDepth.

DEL: Deletion, DUP: Duplication, b: base pair, Kb: Kilobase pair, Mb: Megabase pair, HTZ: Heterozygous, HEM: Hemizygous, aCGH: array-comparative genomic hybridization, MLPA: Multiple Ligation Probe Amplification, GAP-PCR: GAP-Polymerase Chain Reaction, STR: Short Tandem Repeats, SNV: Single Nucleotide Variant, SNP: Single Nucleotide Polymorphism, n/a: not applicable.
