# Supplementary material for: Germline CNV Detection through Whole-Exome Sequencing (WES) Data Analysis Enhances Resolution of Rare Genetic Diseases
Source: Genes (Basel). 2023 Jul 21;14(7):1490. doi: 10.3390/genes14071490 (PMC10379589; doi:10.3390/genes14071490)
Supplement: Supplementary file 1 [file genes-14-01490-s001.zip › Table S2.pdf]

**Table S2.** Characteristics of all the CNVs identified by ExomeDepth in 454 samples.

| | **Deletions** | **Duplications** |
|---|---|---|
| **Number of CNVs** | 69912 | 36547 |
| **Size** | 170b (1 exon) to 13.7 Mb | 320b (1 exon) to …… * |
| **Number of CNVs per individual** | 70 – 188 | 59 – 124 |

*Including two cases of Aneuploidy (Trisomy 21 and Klinefelter). b: base pair, Mb: Megabase pair
