# Supplementary material for: Germline CNV Detection through Whole-Exome Sequencing (WES) Data Analysis Enhances Resolution of Rare Genetic Diseases
Source: Genes (Basel). 2023 Jul 21;14(7):1490. doi: 10.3390/genes14071490 (PMC10379589; doi:10.3390/genes14071490)
Supplement: Supplementary file 1 [file genes-14-01490-s001.zip › Table S3.pdf]

Table S3. List of patients with CNVs detected by ExomeDepth from WES data.

Patient	Main Clinical Features	Type	CNV predicted coordinates (Grch37/hg19)	CNV size	Bayes Factor	Reads Ratio	Genes (Transcript)	Number of coding Genes/Exons (Exon No)	CNV Classification (ACMG Score)	CNV Confirmation method	Inheritance (Additional Comments)	SNV combined with the CNV	Disease (MIM number)
Neurodevelopmental Disorders													
1	GDD, Hypertonia, Seizures	DEL	chr3:9482141_9490314	8,2 Kb	19,1	0.72 (HTZ)	*SETD5* (NM_001080517)	9 Exons (8-16)	LIKELY PATHOGENIC: 0.9 (1A, 2B, 2E, 3A)	n/a	AD	n/a	Intellectual developmental disorder, autosomal dominant 23 (615761)
2	GDD, Dysmorphic features	DEL	chr5:127800418_134002686	6.2 Mb	1080	0.66 (HTZ)	*PPP2CA* + 46 genes	47 Genes	PATHOGENIC: 1 (1A, 2A, 2B, 2C, 2H, 3C, 4L)	n/a	AD	n/a	Neurodevelopmental disorder and language delay with or without structural brain abnormalities (618354)
3	Hypotonia Failure to thrive	DEL	chr12:11463270_14019142	2.6 Mb	477	0.67 (HTZ)	*GRIN2B* + 19 genes	20 Genes	PATHOGENIC: 1 (1A, 2A, 3A, 4L)	MLPA: MRC P410-A2 - *GRIN2B* Deletion	AD, *de novo*	n/a	Intellectual developmental disorder, autosomal dominant 6, with or without seizures (613970)
4	GDD, Hypotonia	DUP	chr2:149220152_149633312	413.2 Kb	51	1.25	*MBD5* (NM_001378120)	9 Exons (6-14)	LIKELY PATHOGENIC: 0.9 (1A, 2B, 2K, 2L, 3A, 4L, 5H)	n/a	AD	n/a	Intellectual developmental disorder, autosomal dominant 1 (156200)
5	Macrocephaly, GDD	DEL	chr17:27834915_27870040	35.1 Kb	32,2	0.6 (HTZ)	*TAOK1* (NM_020791)	7 Exons (14-20)	LIKELY PATHOGENIC: 0.9 (1A, 2B, 2E, 3A)	n/a	AD	n/a	Developmental delay with or without intellectual impairment or behavioral abnormalities (619575)

6	GDD	DUP	chr15:23684691_28566579	4.9 Mb	401	1.21	*MAGEL2, SNRPN, UBE3A, ATP10A, GABRB3 HERC2* + 8 genes	14 Genes	PATHOGENIC: 1 (1A, 2A, 2B, 2H, 3A, 4L)	MS-MLPA: MRC ME028-D1 + STRs	AD (Triplosensitivity), Maternal	n/a	15q11-q13 maternal duplication syndrome (608636)
7	Seizures, GDD	DEL	chr16:29495011_30206548	711.5 Kb	479	0.7 (HTZ)	*PRRT2, QPRT* + 29 genes	31 Genes	PATHOGENIC: 1 (1A, 2A, 2H, 3B, 4L, 4O)	n/a	AD	n/a	Convulsions, familial infantile, with paroxysmal choreoathetosis (602066)
8	Epileptic Encephalopathy, Hypotonia	DEL	chr16:8841965_8851663	9.7 Kb	14,7	0.51 (HTZ)	*ABAT* (NM_020686)	3 Exons (4-6)	LIKELY PATHOGENIC: 0.9 (1A, 2B, 2E, 3A)	Targeted RNA Sequencing: deletion exons 4-6 and Sanger seq for SNV. In trans: Deletion Maternal and SNV Paternal	AR, Compound HTZ with Pathogenic SNV	*ABAT*: c.1153G>T, p.(Asp385Tyr)	GABA-transaminase deficiency (613163)
12	GDD, Hypotonia Seizures	DEL	chr7:72717395_74173168	1.5 Mb	2270	0.53 (HTZ)	*ELN, LIMK1* + 23 genes	25 Genes	PATHOGENIC: 1 (1A, 2A, 2H, 3A, 4L)	MLPA: MRC P245-A2 (*ELN* and *LIMK1* index genes deletion)	AD (Haploinsufficiency), *de novo*	n/a	Williams-Beuren syndrome (194050)
13	GDD	DEL	chrX:153295818_153298008	2.2 Kb	11,8	0.69 (HTZ)	*MECP2* (NM_001110792)	2 Exons (2-3)	PATHOGENIC: 1 (1A, 2B, 2E, 3A, 4L)	MLPA: MRC P015-F2 - *MECP2* Deletion, Exons 2-3	XL, *de novo*	n/a	Rett syndrome (312750)
15	Autism, GDD, Aortic aneurysm, Hypothyroidism	DUP	chr1:146465878_147416212	950.3 Kb	263	1.28	*NBPF14, PRKAB2, FMO5, CHD1L, BCL9, ACP6, GJA5, GJA8, GPR89B*	9 Genes	PATHOGENIC: 1 (1A, 2A, 2H, 3A, 4L, 4N, 4O)	aCGH: arr[GRCh37] 1q21.1q21.2(146507518_147824207)x3	AD (Triplosensitivity)	n/a	Chromosome 1q21.1 duplication syndrome (612475)
16	Epileptic Encephalopathy, Seizures	DEL	chr2:166872248_167334216	462 Kb	116	0.48 (HTZ)	*SCN1A* (NM_006920), *SCN9A, SCN7A*	*SCN1A* 16 exons (1-16), *SCN9A* (whole gene), *SCN7A* 24 exons (3-26)	PATHOGENIC: 1 (1A, 2A, 2B, 2C, 3A, 4L)	MLPA: MRC P137-C1 - *SCN1A* Deletion, Exons 1-16	AD	n/a	Dravet syndrome (607208)

17	GDD, Delayed ability to walk, Myopathy Contractures	DEL	chr8:89179899_97978274	8.8 Mb	638	0.69 (HTZ)	*MMP16, NBN, DECR1* + 35 genes	38 genes	PATHOGENIC: 1 (1A, 2A, 2B, 2D, 2H, 3A, 4L)	aCGH: arr[GRCh37] 8q21.3q22.1(89148404_98017865)x1	AD (Haploinsufficiency)	n/a	n/a
18	GDD, Autism, Aortic aneurysm	DEL	chr15:22833525_23412276	578.8 Kb	465	0.51 (HTZ)	*TUBGCP5, CYFIP1, NIPA2, NIPA1*	4 genes	PATHOGENIC: 1 (1A, 2A, 2H, 3A, 4L, 4N, 4O)	n/a	AD (Haploinsufficiency)	n/a	Chromosome 15q11.2 deletion syndrome (615656)
19	Failure to thrive, Hypotonia GDD	DUP	chr17:16664739_20370783	3.7 Mb	1470	1.38	*RAI1, ATPAF2, B9D1, SREBF1, TOP3A + 48 genes*	53 genes	PATHOGENIC: 1 (1A, 2A, 2E, 2G, 2H, 3C, 4L)	n/a	AD (Triplosensitivity)	n/a	Potocki-Lupski syndrome/Chromosome 17p11.2 duplication syndrome (610883)
Neuromuscular Disorders													
20	Lower limb muscle weakness, Gait abnormalities	DEL	chr9:36227245_36227455	211 b	15,2	0.5 (HTZ)	*GNE* (NM_005476)	1 Exon (7)	LIKELY PATHOGENIC: 0.9 (1A, 2B, 2E, 3A)	n/a	AR, Compound HTZ with Pathogenic SNV	*GNE*: c.1985C>T, p.(Ala662Val)	Nonaka myopathy (605820)
21	Muscle weakness, Myasthenic syndrome	DEL	chr11:47459526_47460482	957 b	16,7	0.51 (HTZ)	*RAPSN* (NM_005055)	2 Exons (7-8)	LIKELY PATHOGENIC: 0.9 (1A, 2B, 2E, 3A, 4E, 5H)	n/a	AR, Compound HTZ with Pathogenic SNV	*RAPSN*: c.264C>A, p.(Asn88Lys)	Myasthenic syndrome, congenital, 11, associated with acetylcholine receptor deficiency (616326)
22	Elevated circulating creatine kinase concentration, Sex reversal	DEL	chrX:31950355_32305635	355.3 Kb	87	0 (HEM)	*DMD* (NM_004006)	2 Exons (44-45)	LIKELY PATHOGENIC: 0.9 (1A, 2B, 2E, 3A, 4L)	MLPA: MRC P034-B2 and P035-B1 - *DMD* Deletion, Exons 44-45	XL	n/a	Becker muscular dystrophy (300376)

23	Difficulty walking, Gait disturbance, Ptosis	DEL	chr19:38987608_38993136	5.5 Kb	22	0.54 (HTZ)	*RYR1* (NM_000540)	5 Exons (43-47)	LIKELY PATHOGENIC: 0.9 (1A, 2B, 2E, 3A, 4E, 5H)	n/a	AR, Compound HTZ with Pathogenic SNV	*RYR1*: c.1250T>C, p.(Leu417Pro)	Minicore myopathy with external ophthalmoplegia (255320)
24	Generalized neonatal hypotonia Neonatal asphyxia	DEL	chrX:149761077_149818374	57.3 Kb	78,9	0 (HEM)	*MTM1* (NM_000252)	9 Exons (2-10)	PATHOGENIC: 1 (1A, 2C, 2E, 3A)	MLPA: MRC P309 Analysis - *MTM1* Deletion, Exons 2-10	XL	n/a	Myopathy, centronuclear, X-linked (310400)
Renal Disorders													
26	Stickler syndrome /Renal disorder	DEL	chr6:491126_1624775	1,1 Mb	32	0.62 (HTZ)	*FOXC1, FOXF2, FOXQ1, GMDS, EXOC2, HUS1B*	6 Genes	PATHOGENIC: 1 (1A, 2A, 2B, 2C, 2H, 3A, 4L)	aCGH: arr[GRCh37] 6p25.3(510688_1671743)x1	AD	n/a	Axenfeld-Rieger syndrome, type 3 (602482)
Metabolic Disorders													
27	Hypertransaminasemia, Weight loss	DEL	chr17:34842545_36293050	1,5 Mb	507	0.65 (HTZ)	*HNF1B* + 14 genes	15 Genes	PATHOGENIC: 1.0 (1A, 2A, 2H, 3A, 4L, 4O)	MLPA: MRC P463-A2- *HNF1B* Deletion	AD, *de novo*	n/a	Renal cysts and diabetes syndrome (137920)
28	Neonatal Jaundice	DEL	chr12:21007963_21392123	384.2 Kb	392	0.005 (HOM)	*SLCO1B3* (NM_019844), *SLCO1B1* (NM_006446)	*SLCO1B3* 15 Exons (2-16), *SLCO1B1* (Whole Gene)	LIKELY PATHOGENIC: 0.9 (1A, 2A, 2B, 2D, 3A, 4L, 4N, 4O)	n/a	AR	n/a	Hyperbilirubinemia, Rotor type, digenic (237450)
29	Abnormal circulating apolipoprotein concentration	DEL	chr5:133956623_133959709	3.1 Kb	15,5	0.32 (HTZ)	*SAR1B* (NM_016103)	2 Exons (2-3)	LIKELY PATHOGENIC: 0.9 (1A, 2B, 2E, 3A)	STRs (Deletion Maternal) + Sanger seq for SNV (SNV Paternal)	AR, Compound HTZ with Pathogenic SNV	*SAR1B*: c.554G>T, p.(Gly185Val)	Chylomicron retention disease (246700)
30	Hypoglycemia	DEL	chr9:97401423_97401592	170 b	24,9	0.52 (HTZ)	*FBP1* (NM_000507)	1 Exon (1)	PATHOGENIC: 1 (1A, 2B, 2E, 3A, 4L)	GAP-PCR: *FBP1* Deletion: chr9:97396231_97401641 (5410bp)	AR, Compound HTZ with Pathogenic SNV (seemed HOM)	*FBP1*: c.170+4A>G	Fructose-1,6-bisphosphatase deficiency (229700)

31	Posterior Embryotoxon, Jaundice, Hyperbilirubinemia, Intrahepatic Cholestasis	DEL	chr18:55020078_56892966	1.9 Mb	814	0.52 (HTZ)	*ATP8B1, ST8SIA3, ONECUT2, FECH, NARS1, NEDD4L, ALPK2, MALT1, ZNF532, SEC11C, GRP*	11 Genes	PATHOGENIC: 1 (1A, 2A, 2H, 3A, 4L)	aCGH: arr[GRCh37] 18q21.31q21.32(54919098_56963819)x1	AD/AR	n/a	Cholestasis, intrahepatic, of pregnancy, 1 (147480) / Cholestasis, progressive familial intrahepatic 1 (211600)
Ocular/Auditory Disorders													
32	Retinitis Pigmentosa	DEL	chr1:216138660_216270555	131,9 Kb	53	0.66 (HTZ)	*USH2A* (NM_206933)	16 Exons (22-37)	PATHOGENIC: 1 (1A, 2B, 2E, 3A, 4L)	MLPA: MRC P362-A3 - *USH2A* Deletion, Exons 22, 24, 26, 28, 30, 32, 34	AR, Compound HTZ with Pathogenic SNV	*USH2A*: c.10712C>T, p.(Thr3571Met)	Usher syndrome, type 2A (276901)
33	Choanal atresia, Microcephaly, Hearing impairment	DEL	chr17:42929777_42937911	8.1 Kb	67,7	0.56 (HTZ)	*EFTUD2* (NM_004247)	10 Exons (17-26)	PATHOGENIC: 1 (1A, 2B, 2E, 3A, 4E)	n/a	AD	n/a	Mandibulofacial dysostosis, Guion-Almeida type (610536)
Congenital anomalies/Syndromic Disorders													
34	Trigonocephaly, Hypotonia, GDD, Thin corpus callosum, Hypertelorism, Carp-shaped mouth	DEL	chr11:120531028_134257553	13.7Mb	4120	0.53 (HTZ)	*HEPACAM, PUS3, ROBO4, FLI1* + 101 genes	105 Genes	PATHOGENIC: 1 (1A, 2A, 2H, 3C, 4L)	n/a	AD (Haploinsufficiency)	n/a	Jacobsen syndrome (147791)

35	Tall stature, Marfan like	DUP	chrX:200855_155240074	155 Mb	5497	1.8	Additional X chromosome	834 Genes	PATHOGENIC: 1 (1A, 2A, 2G, 2H, 3C, 4L)	Conventional Karyotype	Additional X chromosome (Aneuploidy)	n/a	Klinefelter syndrome - 47,XXY
Dermatological Disorders													
36	Ichthyosis	DEL	chrX:6968337_8434424	1.5 Mb	238	0.53 (HTZ)	*STS, PNPLA4, PUDP, VCX, VCX2, VCX3B*	6 Genes	PATHOGENIC: 1 (1A, 2A, 3A, 4E, 4L, 4N, 4O)	n/a	XL	n/a	Ichthyosis, X-linked (308100)
Others Disorders													
37	Primary ciliary dyskinesia	DEL	chrX:105066840_106486528	1.4 Mb	542	0.00873 (HEM)	*PIH1D3, TBC1D8B, SERPINA7* +9 genes	12 Genes	LIKELY PATHOGENIC: 0.9 (1A, 2A, 2H, 3A, 4E)	aCGH: arr[GRCh37] Xq22.3(105088238_106606637)x0	XL	n/a	Ciliary dyskinesia, primary, 36, X-linked (300991)
38	Rhabdoid tumors	DEL	chr22:22893189_24177119	1.3 Mb	257	0.67 (HTZ)	*SMARCB1* + 16 genes	17 Genes	PATHOGENIC: 1 (1A, 2A, 2G, 2H, 3A, 4L, 4N)	MLPA: MRC P258-C2 - *SMARCB1* Deletion	AD, *de novo*	n/a	Schwannomatosis 22q11.22-q11.23 (162091)
39	Severe Neutropenia	DEL	chr1:154247426_154248177	752 b	82,9	0 (HOM)	*HAX1* (NM_006118)	4 Exons (4-7)	LIKELY PATHOGENIC: 0.9 (1A, 2B, 2E, 3A)	n/a	AR	n/a	Neutropenia, severe congenital 3, autosomal recessive (610738)
40	Fanconi anemia	DEL	chr16:89818649_89883044	64.4 Kb	58	0.6 (HTZ)	*FANCA* (NM_000135)	30 Exons (1-30)	PATHOGENIC: 1 (1A, 2B, 2C, 3A, 4L, 4O)	n/a	AR, Compound HTZ with Pathogenic SNV (seemed HOM)	*FANCA*: c.82G>T, p.(Gly28*)	Fanconi anemia, complementation group A (227650)

GDD: Global Developmental Delay, ADHD: Attention deficit-hyperactivity disorder, DEL: Deletion, DUP: Duplication, b: base pair, Kb: Kilobase pair, Mb: Megabase pair, HOM: Homozygous, HTZ: Heterozygous, HEM: Hemizygous, aCGH: array-comparative genomic hybridization, MLPA: Multiplex Ligation dependent Probe Amplification, GAP-PCR: GAP-Polymerase Chain Reaction, STR: Short Tandem Repeats, SNV: Single Nucleotide Variant, AD: Autosomal Dominant, AR: Autosomal Recessive, XL: X-linked, n/a: not applicable.
