# Supplementary material for: Germline CNV Detection through Whole-Exome Sequencing (WES) Data Analysis Enhances Resolution of Rare Genetic Diseases
Source: Genes (Basel). 2023 Jul 21;14(7):1490. doi: 10.3390/genes14071490 (PMC10379589; doi:10.3390/genes14071490)
Supplement: Supplementary file 1 [file genes-14-01490-s001.zip › Table S4.pdf]

Table S4. Whole chromosomal duplications detected by ExomeDepth in two patients referred for WES.

Patient	Karyotype	Disease	ExomeDepth predicted coordinates (Grch37/hg19)	CNV size	Reads Ratio
279	47,XX,+21	Down syndrome (Trisomy 21)	DUPLICATION: chr21:10952913_48084286	37,1 Mb	1.31
35	47,XXY	Klinefelter syndrome	DUPLICATION: chrX:200855_155240074	155 Mb	1.8

Mb: Megabase pair
